# Decoding polyamine–ethylene dynamics: synergies at synthesis and signalling levels during osmotic stress and ripening

**DOI:** 10.3389/fpls.2026.1818877

**Published:** 2026-05-28

**Authors:** Shalu Gupta, Krishan Kant, Navneet Kaur, Parnika Jindal, Magda Pál, M. Naeem, Akbar Ali

**Affiliations:** 1 Plant Physiology and Biochemistry Lab, Department of Botany, Faculty of Science, Dayalbagh Educational Institute (Deemed to be University), Agra, Uttar Pradesh, India; 2 Agricultural Institute, Centre for Agricultural Research, Hungarian Research Network (HUN-REN), Martonvásár, Hungary; 3 Plant Physiology Section, Department of Botany, Aligarh Muslim University, Aligarh, Uttar Pradesh, India

**Keywords:** ethylene, polyamines, regulation and sustainable agriculture, ROS, SAM (S-adenosylmethionine)

## Abstract

Amid climate uncertainty, agriculture faces numerous challenges, including drought, salinity, hypoxia, heat, cold, osmotic, biotic stresses, and, in turn, oxidative stress, in its struggle to secure global food supplies. Plant development is influenced by numerous internal and external factors that collectively regulate the entire life cycle of a plant and, thus, yield. Ethylene (ETH) and polyamines (PAs) are multifunctional regulatory compounds. Their interaction plays a significant role under osmotic stress and fruit ripening, both synergistically and antagonistically. Although both of them are produced from the same precursor, S-adenosylmethionine (SAM), their signalling pathways differ and induce distinct physiological effects that are often antagonistic. Putrescine, spermidine, and spermine are the most abundant PAs that are typically linked to stress resilience, membrane stability, antioxidative responses, and cellular defence. ETH, on the other hand, exhibits two distinct characteristics: at moderate levels, it functions as a ripening hormone stress signal to initiate adaptive responses, but at higher or prolonged stress levels, it promotes senescence and inhibits growth. This review article discusses SAM as the common linking molecule between the overlapping ETH and PA biosynthesis, ETH–PA interaction under osmotic stress, as well as their signalling and regulatory crosstalk in ripening, and how ROS are generated and mitigated by ETH and PA application in a stressful environment. We discuss recent advances in this subject, highlight some unanswered questions, and provide a roadmap for increasing productivity and unleashing climate-smart farming.

## 1 Introduction

Fluctuating environmental conditions can decrease the growth and productivity of plants by triggering the effects of different stress factors (Son and Park, 2022; Ali et al., 2024). Osmotic stress, caused by abiotic stresses such as drought, salinity, and heat, affects plant cellular homeostasis. Plants have specific receptors by which they sense a stressful environment and cascade different, complex signalling pathways that provide resilience against stress. According to Gupta et al. (2024), signalling molecules, plant growth regulators (PGRs), and plant hormones play an important role in these cascades. Phytohormones, such as ethylene (ETH), are capable of regulating plant growth and development at extremely low concentrations under both favourable and unfavourable environmental conditions (Waadt et al., 2022). However, PGRs, like polyamines (PAs), are more prevalent in plants as they regulate biological effects at millimolar concentration (mM), suggesting that they may not have a truly hormonal function (Zahid et al., 2023; Maurya et al., 2025). Mutual interaction between PAs and ETH is not limited only to their synthesis antagonism; their crosstalk exists at signalling and transcriptional levels, too. Focusing on this multilevel connection, in the present review, we discuss how the fine-tuned ETH–PA relationship is vital for the growth and development of plants. Understanding this dynamic interface offers promising opportunities for developing stress-tolerant plants.

ETH is the only gaseous plant hormone that is essential for regulating several diverse plant processes, for instance, seed dormancy and germination, root growth and nutrient acquisition, leaf abscission, fruit ripening, senescence, shade avoidance, and different physiological and molecular mechanisms (such as photosynthesis or signalling, even epigenetics) under stressless conditions and stress acclimation (Wang et al., 2025; Nazir et al., 2024; Pierik et al., 2004). It serves as a crucial mediator by combining endogenous developmental cues with external environmental signals (Iqbal et al., 2017; Dubois et al., 2018; Wang et al., 2025). Although, originally, ETH was classified as a plant hormone that controls senescence, as a Janus-faced molecule, it also has some stimulatory effects depending on its concentration, the plant species, and the given biological process (Pierik et al., 2006).

**Figure 1:**
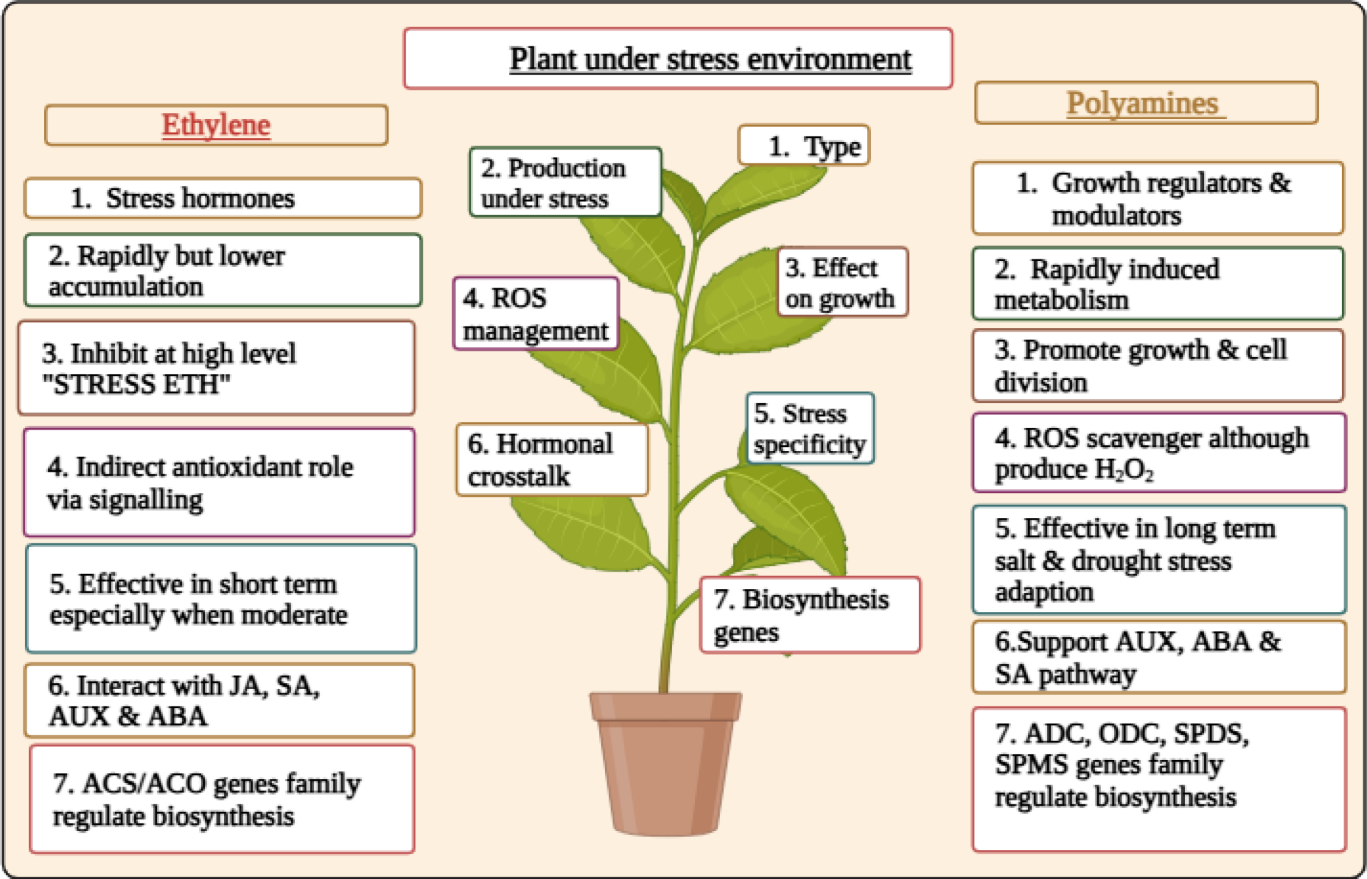
Comparative overview of ETH’s and PA’s roles, functions, and action mechanisms under stressful environments in plants. ETH, ethylene; PA, polyamine; JA, jasmonic acid; SA, salicylic acid; ABA, abscisic acid; ROS, reactive oxygen species; AUX, auxin; ADC, arginine decarboxylase; ODC, ornithine decarboxylase; SPDS, spermidine synthase.

Synergistic and antagonistic relationships between ETH and other plant hormones make it possible to exert their complex effects at the enzymatic, metabolite, and gene expression levels. Promoting the effect of ETH on auxin (AUX) synthesis and transport increases cell elongation and root growth (**Figure 1**) (Swarup et al., 2007; Růžička et al., 2007). Meanwhile, ETH antagonises abscisic acid (ABA)-induced stomatal closure and dormancy (An et al., 2025; Sun et al., 2020). Also, a negative signalling crosstalk exists between salicylic acid (SA) and ETH/jasmonic acid (JA), especially during biotic stress, as different defence signalling and mechanisms are required to be induced upon biotrophic and necrotrophic pathogen attacks (Li et al., 2019). ETH activates the gene expression of certain antioxidant enzymes; thus, it can also induce stress tolerance (Hu et al., 2016). By inducing certain catabolic enzymes, it stimulates the conversion of stored starch into soluble sugars and, in turn, for example, induces germination or maintains osmotic potential under stress conditions (Sun et al., 2020; Cui et al., 2015). The accumulation of phenols, flavonoids, and anthocyanins, also induced by ETH, in addition to stress protection, affects the pigmentation of ripening fruits (Watkins et al., 2014; Dong et al., 2020).

PAs are aliphatic, polycationic amines that, as versatile compounds, regulate numerous physiological and biochemical processes in plants, such as embryogenesis, root formation, flower induction, fruit development, and ripening (He et al., 2022; Gupta et al., 2024). In addition, they are involved in many fundamental cellular processes, such as cell division and elongation, transcription, translation, photosynthesis, stomatal closure, membrane stability, and even programmed cell death (PCD) (Chen et al., 2019; Blázquez, 2024). The overall PA content and its distribution within the plant differ, depending on the plant tissue/organ, the developmental phase, and the presence or absence of stress factors. Generally, its level is higher in young, rapidly dividing and developing parts (meristems, root tips, flowers, or germinating seeds) whilst declining in later phases, as a sign of senescence (Abbasi et al., 2017; Gupta et al., 2024; Ribeiro et al., 2024). PAs occur in free or conjugated forms, either covalently or non-covalently (Seifikalhor et al., 2020). They covalently bind to phenolic compounds such as ferulic acid, perchloric acid, coumaric acid, caffeic acid, cinnamic acid, etc. Covalently bound PAs have higher antioxidant capacity but less mobility compared to the free form and are not quickly mobilisable storage forms, but influence the defence system not only locally but also through broader plant interaction (Chen et al., 2019; Chen B. X. et al., 2023; Pál et al., 2021). The mode of action of PAs may be more complex than that of ETH. As its synthesis and catabolism are linked to several other protective or signalling compounds (NO, H_2_O_2_, proline, phytochelatins) (Pál et al., 2017b; Pál et al., 2018a; Pál et al., 2021), it is difficult to imagine a cellular process that would not have at least an indirect effect (Tan et al., 2022). For example, PAs act directly as osmoprotectants, metal chelators, and antioxidants and also show an effect on gene expression and even DNA methylation (Marco et al., 2011; Pál et al., 2018a; Zhong et al., 2020; Majláth et al., 2025). Accordingly, a small change in PA content/metabolism influences the response of the plant under optimal and stressful environments (Wu et al., 2022; Mamani-Huarcaya et al., 2023; Li et al., 2024).

Hormonal crosstalk between plant hormones and PAs is also well-studied in different plant species. A positive relationship between PAs and SA, in addition to a positive feedback connection between PAs and ABA, has also been demonstrated (Pál et al., 2018b; Szalai et al., 2017). Synergistic AUX–PA interaction in the control of root inductive processes has also been reported (Hausman et al., 1995). While it was also found that the SA-induced spermine (spm) accumulation is controlled by ETH, in addition, salt stress-induced Spm accumulation also depended on ETH signalling in tomato (Takács et al., 2021, 2022). Under osmotic stress, the interaction between ETH and PAs plays an important role in the survival of plants, as PAs show a protective role in maintaining the ion balance, scavenging ROS, and providing membrane stability. On the other hand, ETH regulates growth inhibition and senescence. In the context of fruit ripening, ETH works as a central regulator by regulating processes, such as softening, genes that provide colour and aroma (Liu et al., 2024). In contrast, PAs delay ripening through stabilising cellular structure and antagonising ETH function (Takács et al., 2021).

These results indicate that although PAs are not classical plant hormones, they are important complements of the plant hormone system and essential modulators of hormonal interactions in plant growth, development, and stress tolerance.

Recent discoveries have been underway to uncover the molecular interactions between PAs and ETH, which are often linked to a biochemical ‘tug of war’ with significant developmental complications. This review provides a mechanistic understanding of how these regulatory molecules influence the synthesis and signalling of each other and how these processes accompany them. Highlighting these connections can be used to modify plants’ resistance to abiotic stress.

## 2 S-adenosylmethionine: a shared node in ethylene and polyamines biosynthesis

In plants, S-adenosylmethionine (SAM) is a crucial methyl- and aminopropyl-donor and metabolic intermediary in the Yang cycle, thus serving as an essential component in the production of ETH and PAs (Sauter et al., 2013). Three enzymes are required to generate ETH (Riyazuddin et al., 2020; Maurya et al., 2025). A methionine (sulphur-containing amino acid) is first transformed by the enzyme SAM synthetase (SAMS) into S-adenosyl methionine (SAM), the active form of methionine and a key precursor molecule. Following this, ACC synthase (ACS) converts SAM into 1-aminocyclopropane-1-carboxylic acid (ACC), which is then oxygenated to ETH by ACC oxidase (ACO). As by-products, CO_2_ and cyanide are released (Adams and Yang, 1981; Pattyn et al., 2021). In a sequence of enzymatic processes, the Yang cycle converts the 5-methylthioadenosine, which is produced as a by-product of ACS, back to methionine (Pommerrenig et al., 2011). Overall, ACS and ACO enzymes tightly regulate ETH biosynthesis at both the transcriptional and post-transcriptional levels (Zhao et al., 2021), where the ACS enzyme limits the rate of ETH production (Xu et al., 2021).

Conversely, putrescine (Put) is the first PA that is produced when arginine decarboxylase (ADC) (chloroplast localised) or ornithine decarboxylase (ODC) (cytoplasma localised) decarboxylate L-arginine or L-ornithine, respectively (Walters, 2003; Napieraj et al., 2023). However, in *Arabidopsis thaliana*, Put is synthesised from arginine by the paralogues of ADC, i.e., AtADC1 and AtADC2. These enzymes are localised in both the cytosol and the chloroplast. In addition to their subcellular localisation, ADC proteins have been reported to form homodimers in the cytosol as well as heterodimers of AtADC1/AtADC2 proteins (Maruri-López and Jiménez-Bremont, 2017). After that, in the cytoplasm, spermidine synthase (SPDS) converts Put into spermidine (Spd), which spermine synthase (SPMS) transforms into spermine (Spm) (Vuosku et al., 2018). The enzyme S-adenosylmethionine decarboxylase (SAMDC) transforms SAM into decarboxylated SAM (dcSAM), which can be used in both of these conversions as an aminopropyl donor (Napieraj et al., 2023; Gupta et al., 2024). The catabolism of PAs is also important, as various small molecular weight protective and signalling compounds such as GABA, H_2_O_2_, and β-alanine are produced as by-products during the oxidation catalysed by PA oxidases (CuAOs and PAOs) (Benkő et al., 2022; Gupta et al., 2024).

As the biosynthetic pathways of ETH and PAs are interconnected, the production of one of them can affect the other. According to Sauter et al. (2013), both ETH and PAs share a common precursor in SAM; hence, when SAM availability is low, the biosynthesis of ETH and PAs may compete for this substrate. For instance, PA content increases when the synthesis of ACC, a precursor of ETH, is inhibited. On the other hand, ACC and ETH concentrations rise when PA production is inhibited, suggesting that when one SAM-dependent pathway is blocked, the other is stimulated (Rauf et al., 2021). The distribution of SAM for the synthesis of ETH or PA in tomato fruits was examined using ^14^C-radiolabelled methionine (Lasanajak et al., 2014). According to the findings, when the ETH level rose in the later stages of ripening, the PA synthesis decreased. Nevertheless, this decrease in PA level was restored by transgenic expression of the yeast *ySAMDC* gene, which had no effect on ETH concentrations. These findings imply that SAM’s cellular flow is distributed according to its need for competing routes for the synthesis of PAs or ETH (Lasanajak et al., 2014). The *Arabidopsis amiR:ADC* line, in which the *ADC1* and *ADC2* genes are silenced, exhibited reduced polyamine levels, resulting in stunted growth and delayed flowering, which were reversed upon the application of exogenous PAs, confirming the essential role of PAs (Sánchez-Rangel et al., 2016*).* Overexpression of the cucumber gene *CsSAMS1* in tobacco can lower the Put level, whilst increases Spd and Spm levels, as well as ETH content, and provides salt stress tolerance of tobacco (Zhu et al., 2021). According to Montilla-Bascón et al. (2017), *Hordeum vulgare* wild type (WT) exhibited a noteworthy rise in total PA content during drought, but this was lower than that of transgenic plants (UHb plants). UHb plants are overexpressing Non-symbiotic Class 1 Hemoglobin Gene (Hb1), which results in lower NO content, and in turn, higher levels of PAs but lower ETH production. In WT plants under drought, Put levels were increased by the upregulation of *ADC*; however, *SAMDC* was downregulated, whilst *ACS* was upregulated. In other words, the conversion of Put decreased to higher PAs, Spd, and Spm, and methionine is diverted to the ETH biosynthesis pathway rather than the PA biosynthesis pathway. In UHb plants, large amounts of Put in the early period of drought converted to higher PAs, which help them adapt to stress.

**Figure 2:**
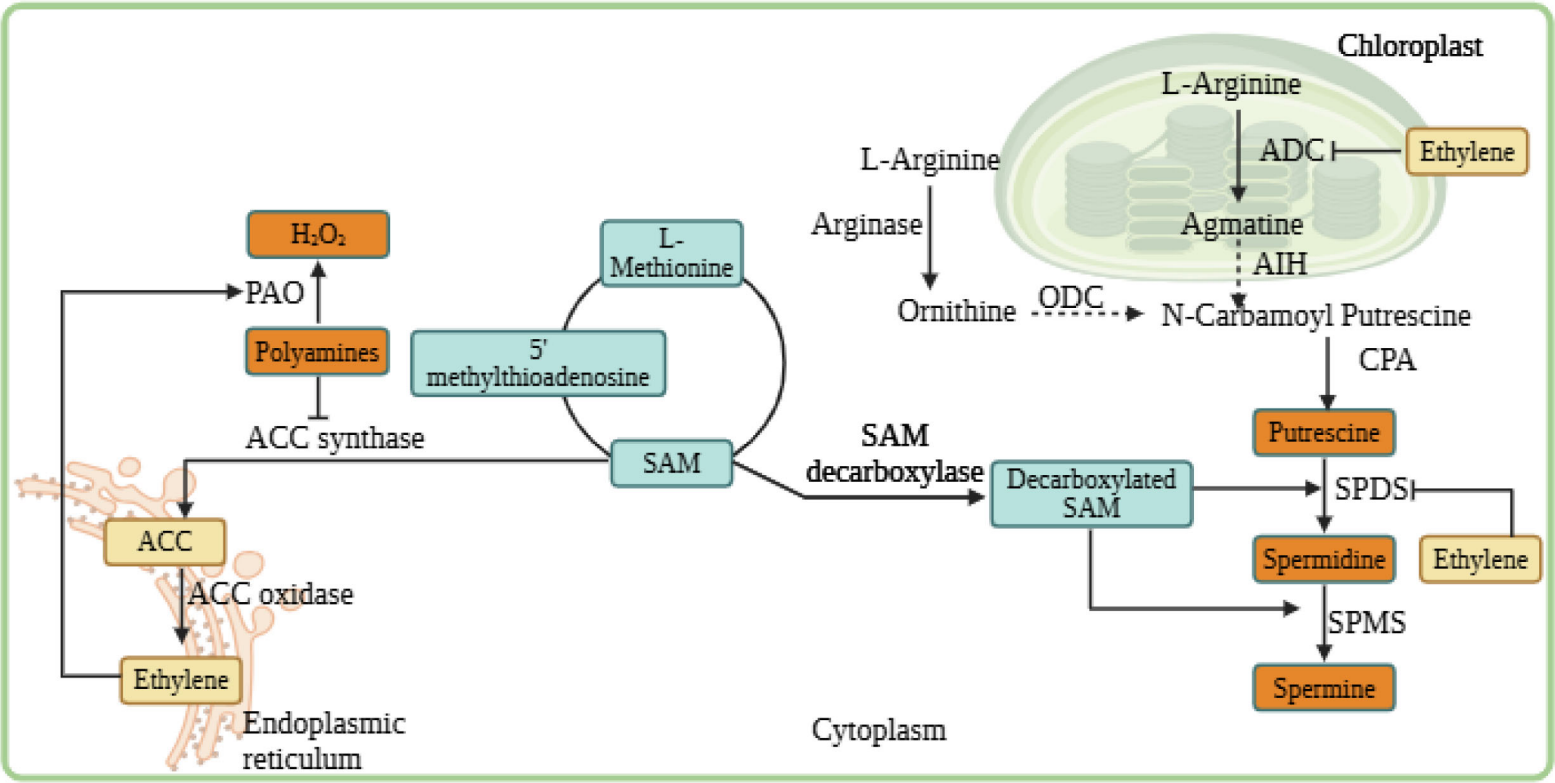
A schematic figure showing how SAM is interlinked with PA and ETH biosynthesis and how ETH and PAs influence the metabolism of each other. PAO, polyamine oxidase; ACC, 1-aminocyclopropane-1-carboxylic acid; SAM, S-adenosylmethionine; ODC, ornithine decarboxylase; ADC, arginine decarboxylase; AIH, agmatine iminohyrolase; CPA, N-carbamoylputrescine amidohydrolase; SPDS, spermidine synthase; SPMS, spermine synthase.

In summary, fine-tuning of ETH–PA synthesis is an important part of stress tolerance. Under optimal conditions or during stress adaptation, in general, the synthesis of PAs is dominant, but under prolonged or serious stress conditions, a shift from PA to ETH synthesis results in a further decrease in PA level, due to the ETH-induced PA catabolism, which produces H_2_O_2_ and elevates oxidative stress. A dynamic model of the ETH–PA action mechanism has been provided by Freitas et al. (2018). It was found that different strategies were applied depending on the level of salt stress tolerance in maize. In a salt-tolerant genotype, a single, early ETH accumulation peak was responsible for the induction of CuAO-mediated H_2_O_2_ production and signalling, which activate plant stress defence responses. However, later on, ETH synthesis and PA catabolism were inhibited, whilst *ADC* expression increased and PAs accumulated, with the latter having an important role in stress acclimation. In contrast, in the sensitive genotypes, the first ETH production peak did not induce the catabolism of PAs by diamino oxidase, but in the second phase, it resulted in a burst of ETH production (‘stress ETH’), which could inhibit *ADC* but activate *CuAO* expression, inducing PA depletion and harmful stress effects. The complex regulatory crosstalk between the synthesis and/or catabolism of ETH and PAs was also confirmed by the studies, where it was also reported that Put could decrease ETH synthesis by the inhibition of ACS (Yu et al., 2016), whilst Spd could decrease ETH synthesis by the inhibition of ACS and ACO, both at the enzymatic and gene expression levels (Li et al., 2013). In contrast, ETH enhanced the expression of PAOs (Benkő et al., 2022) (**Figure 2**).

## 3 Signal transduction and molecular interaction: the converging roles of polyamines and ethylene

Plants continuously synthesise PAs and ETH throughout their lives, although the rate and reason for their synthesis and their role vary depending on developmental stages and environmental factors. ETH controls plant growth and development under ideal and challenging conditions (Asgher et al., 2018; Riyazuddin et al., 2020). However, in general, ETH synthesis is more prevalent during senescence, ripening, and stress reactions. ETH plays a major role in cell division by showing a positive effect on cell expansion. Moreover, it acts on microtubule orientation and EXPANSIN gene family (Polko et al., 2012; Dubois et al., 2018). On the other hand, PAs are created in greater quantities during early development and vigorous cell division, and functioning as a senescence inhibitor, they have the ability to reverse a variety of stress responses (Poór et al., 2019). However, the level of PAs is not always correlated with the level of stress or stress tolerance, suggesting that the intensity of their metabolism is more important than the content itself (Pál et al., 2021).

**Figure 3:**
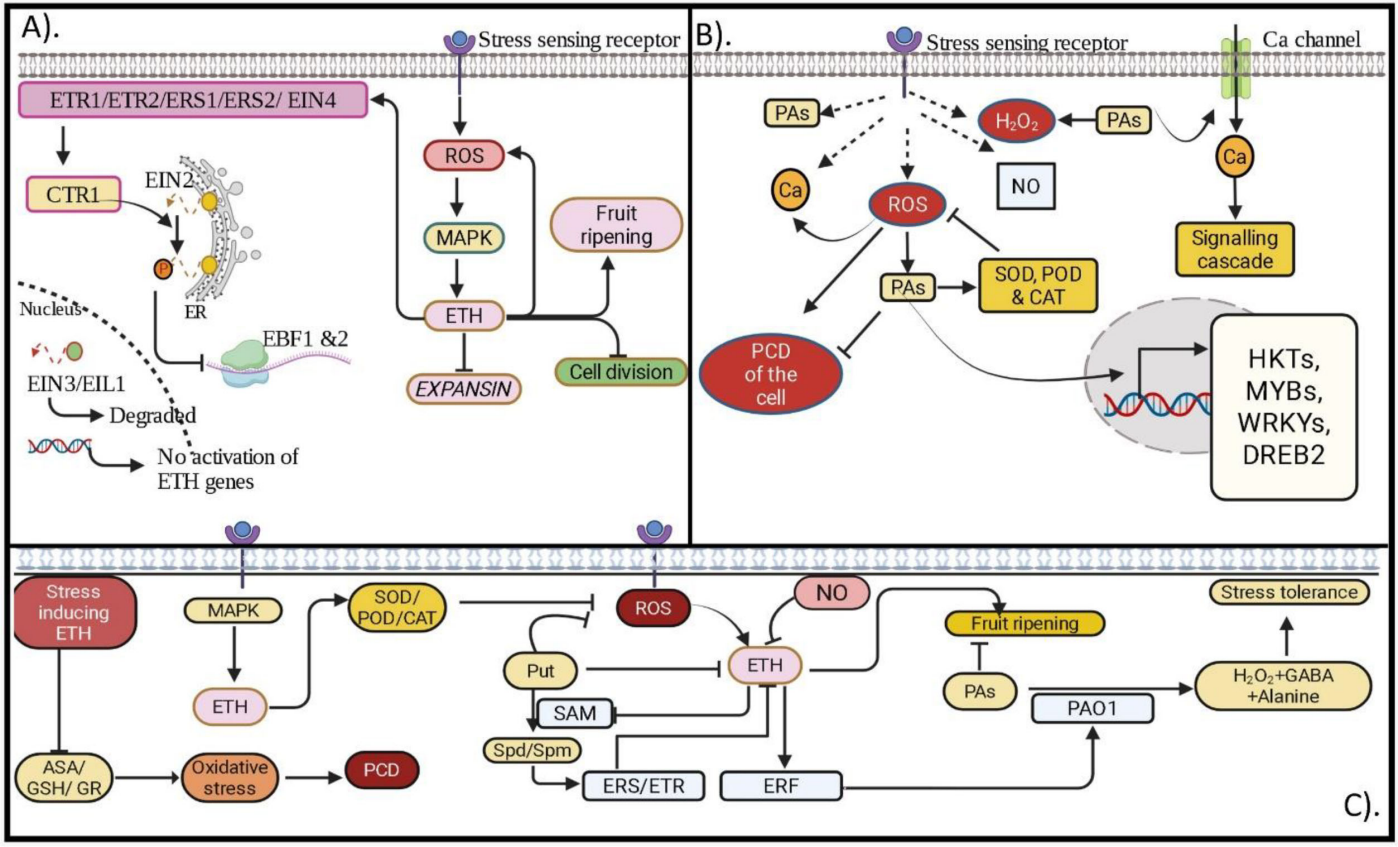
**(A)** Ethylene (ETH) signalling; **(B)** polyamine (PA) signalling; and **(C)** the interaction of ETH–PA signalling under osmotic stress and ripening. Ethylene receptors (ETR1, ETR2, ERS1, ERS2, EIN4); CTR, CONSTITUTIVE TRIPLE RESPONSE1; EIN2, ETHYLENE INSENSITIVE2; MAPK, mitogen-activated protein kinase; NO, nitric oxide; ASA/GSH/GR, ascorbate, glutathione, and glutathione reductase; PCD, programmed cell death; Spd, spermidine; Spm, spermine; SOD/POD/CAT, superoxide dismutase/peroxidase/catalase; ERF113, ethylene receptor factor; PAO1, polyamine oxidase; GABA, gamma-aminobutyric acid.

ETH is produced at the site of action and readily diffuses to neighbouring cells and synthesise in plants under environmental stressors, including drought, salinity, and flood stress (Husain et al., 2020). ETH biosynthesis and signalling involve several processes with distinct regulatory levels that control both its production and the corresponding responses (Fatma et al., 2022). In *Arabidopsis*, ETH is sensed by the ETHYLENE RESPONSE1 (ETR1 and ETR2), ETHYLENE RESPONSE SENSOR1 (ERS1 and ERS2), and ETHYLENE INSENSITIVE4 (EIN4) receptor family. They are found at the membranes of the endoplasmic reticulum (ER) and Golgi apparatus (Dong et al., 2010; Binder, 2020; Fatma et al., 2022; Hao et al., 2025), act negatively in ETH signalling, and are associated with two-component histidine kinase receptors (Chang et al., 1993; Sakai et al., 1998). When ETH is not present, ETH receptors trigger the Raf-like Ser/Thr protein kinase CONSTITUTIVE TRIPLE RESPONSE1 (CTR1) (Guo and Ecker, 2004; Huang et al., 2003), which phosphorylates the C-terminal end of the ER-localised membrane protein EIN2 (EIN2-CEND), thereby blocking it. Parallel with this, the EBF1 and EBF2, F-box proteins containing the SCF-E3 ubiquitin ligase complex, target the EIN3, EIL1, and EIL2 transcription factors, which together with similarly ubiquitinated, phosphorylated EIN2, are also broken down. Therefore, there is no activation of ETH-stimulated transcriptional responses (Binder, 2020; Wang et al., 2025). When ETH is present, ETH attaches to its receptors, shutting down; CTR1 is rendered inactive; and EIN2 is cleaved and dephosphorylated and its C-terminus (EIN2-CEND) is released. The C-terminus translocates into the cytosol (migrating to P-bodies) and the nucleus. By binding directly or indirectly to the 3′-untranslated regions (3′-UTRs) of EBF1 and EBF2 transcripts, EIN2-CEND suppresses their translation in the cytosol and directs them to P-bodies (Li et al., 2015). EIN2-CEND either directly or indirectly raises EIL1 and EIN3 activity in the nucleus. ETH stabilises the EIN3/EIL1 transcription factors and controls the transcription of target genes that respond to ETH. ETH biosynthesis and signalling under stress are a highly controlled and dynamic system. The initial few hours of stress cause a rise in ROS generation, which in turn activates mitogen-activated protein kinase (MAPK) and raises ETH synthesis (Valluru et al., 2017). During the first, immediate ETH accumulation driven by the induced ACC, rapid responses are activated to initiate protective mechanisms, for example upregulating antioxidant machinery and lessening oxidative stress. Meanwhile, after a few days of stress, in the second, delayed phase, the sustained excessive accumulation of ETH (due to its autocatalytic synthesis) decreases plant fitness and causes H_2_O_2_ generation, oxidative damage, senescence, and programmed cell death (PCD), indicating a critical role for ETH as well as ROS in plant signalling (Riyazuddin et al., 2020; Yadav et al., 2023). Stress-induced ETH synthesis downregulates the synthesis of ascorbate (ASA), glutathione (GSH), and glutathione reductase (GR), resulting in a weak antioxidant system and further ROS buildup (Ansari et al., 2019). Moreover, increased superoxide dismutase (SOD) and catalase (CAT) transcript levels were detected in the *ein*2 (ethylene-insensitive 2) mutant, suggesting that ETH contributes to suppressing SOD and CAT expression and, consequently, the accumulation of ROS, inhibiting non-enzymatic antioxidants, primarily GSH and α-tocopherol (α-TOC) (Lin et al., 2012). However, in other cases, especially under stress conditions, ETH increased SOD and peroxidase (POD) activities (Zhou et al., 2013; Wang et al., 2020). Multiple regulatory points, such as receptor modulation, MAPK signalling, EIN2 cleavage, and transcription factor activation (EIN3/EIL1), contribute to the intricacy of the ETH pathway (**Figure 3A**).

Several earlier studies demonstrated that PA metabolism changes rapidly in response to abiotic stress, which may initiate several signalling cascades to help adapt to the stressful environment (Gupta et al., 2024; Shao et al., 2022). PAs scavenge ROS as well as produce H_2_O_2_ and NO as the by-products of PA catabolism (Yu et al., 2018; González-Hernández et al., 2022). Additionally, they change H^+^ pumping, increase OH^−^-induced K^+^ efflux, promote plasma membrane Ca^2+^-ATPase activity, and modify and restructure anion and cation conductance at the plasma membrane. All of these actions will control different downstream signalling pathways. H_2_O_2_ can directly mediate several activities in plants, including stomatal closure, as it affects ion channels and regulates the stress-related MAPK cascade (Liu et al., 2023). PA catabolism is as important as its synthesis; CuAOs and PAOs play an essential role in this process. Firstly, PAOs oxidise Spd or Spm, producing H_2_O_2_ and 1,3-diaminopropane (DAP) and 4-Aminobutanal or N-(3-aminopropyl)-4-aminobutanal, respectively. Back conversion of Spm to Spd and Spd to Put also occurs, due to a reaction catalysed by a special group of PAOs, but here, H_2_O_2_ is also produced as a by-product (Cona et al., 2006; Wang et al., 2019; Benkő et al., 2022; Amiri et al., 2024). Meanwhile, CuAOs are responsible for the catabolism of diamines, such as Put. Raziq et al. (2022) stated that Spd treatment effectively mitigated the adverse effects of salinity in tomato seedlings by triggering H_2_O_2_-mediated signalling, which enhances the expression of salt stress-responsive genes (*SlWRKY1*, *SlHKT1*, *SlDREB2*, and *SlMYB102*) and NADPH oxidase/respiratory burst oxidase homologue (RBOH) protein, as well as by boosting detoxification through elevated antioxidant activity and osmolyte (proline) accumulation. PAs themselves act as antioxidants and, together with ETH, mitigate stress by modulating ROS levels through upregulating antioxidant enzyme expression, preventing plants from oxidative damage (Tang and Newton, 2005; Nahar et al., 2016; Li et al., 2018; Raziq et al., 2022; Yadav et al., 2023). Chávez-Martínez et al. (2018) reported that PA depletion, achieved through silencing of *ADC* genes, leads to increased ROS accumulation by disrupting the balance between ROS production and scavenging systems, highlighting the role of PAs in redox homeostasis and plant development. Ahanger et al. (2019) reported that higher PAs, such as Spd and Spm, upregulated the activities of antioxidant enzymes, including GR, SOD, ascorbate peroxidase (APX), and CAT, as well as increased the levels of non-enzymatic antioxidants such as GSH, ASA, and α-TOC, which enhanced osmolyte accumulation, caused better relative water content, strengthened membranes, and improved photosynthetic performance and protection against oxidative damage caused by salt stress in tomatoes. Similarly, under abiotic stress conditions, Put application markedly upregulated the *m*RNA expression of APX, CAT, and SOD, leading to reduced H_2_O_2_ accumulation and decreased lipid peroxidation, whilst improving membrane stability (Kumar et al., 2014). The expression levels of WRKY, MYB, and NAM transcription factors also show significant responses to Spd treatment (Sun et al., 2022; Xing et al., 2023). Transcription factors, like MbWRKY3, 4, and 65, regulate the scavenging of ROS and the expression of stress-responsive genes (*SlMYB102*, *SHKT1*, *SIWRKY*, *SIDREB2*), providing tolerance against drought (Han et al., 2018; Yu et al., 2024). Apart from this, numerous secondary signalling molecules, including calcium (Ca^2+^), nitric oxide (NO), hydrogen sulphide (H_2_S), and hormones, accumulate under abiotic stresses (Mohanta et al., 2018; Gupta et al., 2023) and show crosstalk with ETH and PA. Notably, the intracellular free Ca²^+^ concentration rapidly generates distinct, stimulus-specific patterns in response to external stress signals and PA treatments (Pottosin et al., 2014; Gupta et al., 2023; Zhang et al., 2022; Gupta et al., 2025, 2026). As a small, highly diffusible gaseous molecule, NO functions as an intra- and intercellular messenger in plants. PA treatments have been reported to increase NO content; in addition, the influence of NO on PA synthesis has also been demonstrated (Tripathi et al., 2023). It has been suggested that NO can fill the gap between certain plant responses and effects of PAs (Pál et al., 2015) (**Figure 3B**). In addition, it has also been reported that NO represses the expression of genes involved in ETH biosynthesis and post-translationally (through S-nitrosylation) modifies methionine adenosyl transferase (MAT) activity to reduce the availability of methyl groups required for ETH synthesis (Manjunatha et al., 2012).

Interestingly, Put or Spd treatment (1 mM) in abscising mature olive fruits increased the expression levels of ERS1 and inhibited the ETH signalling pathway (Parra-Lobato and Gomez-Jimenez, 2011). Similarly, Spd treatment (0.1 mM) in peach fruits due to a higher expression of ETR1 and ERS1 led to lower ETH perception and signalling (Ziosi et al., 2006). A complex biosynthetic crosstalk, with positive or negative regulation loops between ETH, NO, and Pas, started to be figured out; however, unraveling the clear picture still needs further investigation (Maurya et al., 2025) (**Figure 3C**). In addition, H_2_O_2_ accumulation can also lead to NO production, and NO can act as a signalling molecule in the signalling pathway of other plant hormones, such as cytokinin, ABA, auxin, cytokinin, and ETH signalling (Neill et al., 2002). Although H_2_O_2_ and NO play major roles in PA signalling, they are not the only signalling components involved signalling.

DNA methylation, a fundamental process of epigenetic regulation, plays a key role in plant development and stress responses. SAM (a universal methyl donor) is synthesised by SAMS and serves as a common link between DNA methylation and the biosynthesis of ETH and PAs. Research suggests that DNA methylation controls a range of developmental processes, such as seed development, root growth, flower opening, senescence, fruit maturation, and flower sex determination (Geng et al., 2026). Hu et al. (2023) observed that upregulation of *AtSAMS* regulates the expression of important floral (*ABCE*) genes through DNA hypomethylation, which leads to an elevated ETH level. Chen X. et al. (2023) reported that *SlSAMS1* gene overexpression in tomato improves salt tolerance by modulating gene expression and DNA methylation. The *SlGI* gene, which is involved in the circadian rhythm pathway, becomes hypermethylated, resulting in less oxidative damage and increased antioxidant enzyme activity.

## 4 Polyamine-ethylene interplay under osmotic stress and during fruit ripening

Several abiotic stress factors, including drought, non-optimal temperature, nutrient deficiencies, salinity, or toxic metals, pose a continual challenge to sessile plants. These environmental stresses create osmotic stress, which creates difficulties in absorbing water and minerals from the soil because of lower water potential. In plants under osmotic stress conditions, a complex cascade of symptoms develops, involving several changes, such as cell dehydration; hence, the turgor pressure of the cell is reduced, resulting in stomatal closure, decreased relative water content, elevated ROS production and oxidative stress, membrane damage and increased cell electrolyte leakage, reduced photosynthetic pigment levels, impaired photosynthesis, shortened root and shoot growth, and ultimately, lower yield. To mitigate the negative impacts of osmotic stress, the crops are experiencing a variety of morphological, structural, physiological, biochemical, and molecular alterations (**Table 1**) (Naseem et al., 2018; Tang et al., 2018; Ma et al., 2022; Vishnupradeep et al., 2022; Zhang et al., 2022; Nicotra et al., 2025); however, in the end, the growth, development, and productivity of the plant decrease (Fahad et al., 2017). Under osmotic stress, plants develop several defensive mechanisms such as hormonal signalling; osmotic adjustment; upregulation of transcription factors such as WRKY, MYB, DREB (dehydration-responsive element binding), CBF (C-repeat binding factor), and ERF (ethylene response factor), bHLH (basic helix-loop-helix); and stress-responsive gene regulation (Hrmova and Hussain, 2021; Yang et al., 2021). Upregulation of these transcription factors controls processes like root architecture alteration, stomatal regulation, activation of antioxidant defence mechanisms, and hormonal balancing (Hrmova and Hussain, 2021; Yang et al., 2021). Upon osmotic stress, ABA-dependent and independent pathways can be activated. For example, in the regulation of stomatal closure, ABA and ETH are in an antagonistic relationship, as ETH inhibits the ABA-induced closure via the regulation of *NtMYB184* in tobacco (Song et al., 2023). Meanwhile, a novel mode of interplay between ETH and ABA was demonstrated in the control of rice growth and development, as inhibition of rice root growth by ETH required ABA action (Ma et al., 2014). However, a significant part of osmotic stress responses is mediated by an ABA-independent pathway, which is associated with the ETH response factor (ERF) as it was found in *MbERF12*-overexpressor *Arabidopsis* (Han et al., 2021). Soil salinity also creates osmotic potential and specific ion toxicity, which reduces the plant’s ability to absorb water and essential minerals such as Ca²^+^ and K^+^, whilst allowing Na^+^ and Cl^-^ ions to enter cells, damaging the cell membrane and disrupting cytosolic metabolism. Osmotic stress caused by salinity negatively affects plants by impairing membrane function, altering cytosolic metabolism, generating ROS, and inhibiting cell growth. In UHb transgenic barley plants, Montilla-Bascón et al. (2017) observed that delayed senescence improved drought tolerance because of the accumulation of PAs over ETH. They observed upregulation of the *SAMDC* gene and downregulation of the *ACS* gene, which reflects the synthesis of higher PAs over the synthesis of ETH. Zhang et al. (2017) also observed the same antagonist behaviour of ETH and PA. They show that mild drought stress can be tolerated by rice because of higher PA but lower ETH accumulation. However, at an increased level of stress, the ETH level rose and the PA level dropped, making the plant susceptible. Chu et al. (2023) found that sensitivity of rice plants to drought stress was accompanied by the accumulation of ETH, ACC, H_2_O_2_, and related enzymes. It is also hypothesised that the PA: ETH ratio is important in nitrogen assimilation of rice grain during grain filling. Xu et al. (2022) found that ETH inhibited amino acid synthesis, whilst PAs enhanced it (Xu et al., 2021). On the other hand, Takács et al. (2022) found that PA and ETH worked in a synergetic way, and ETH plays an essential role in regulating PA metabolism and oxidative stress responses. WT tomatoes have higher levels of Spm and antioxidant enzymes, whilst the ETH receptor-mutant *Never ripe* (*Nr*) tomatoes failed to show these responses. Moreover, elevated PA catabolism was reported in *Nr* plants, leading to oxidative damage, whereas ETH and PAs acted together in WT plants to alleviate salt stress.

**Table 1:** The role of ETH and PAs in abiotic stress management.

Stress type/plant name	ETH response	PA response	Impact on the plant	References
Osmotic (*Glycyrrhiza inflata*)	- Increased ETH production - ROS stimulated ETH production	- Decreased PAs	- Severe stress caused ROS production	Li and Wang (2004)
Drought (*Brassica rapa*)	- Increased ETH production	- Increased Put - Reduced Spd and Spm	- Application of Spd or aminooxyacetic acid (an inhibitor of ETH synthesis) enhanced ribulose 1,5-bisphosphate carboxylase activity by modulating PA levels and decreasing the damage caused by ETH release	Huang et al. (2014)
Aluminum (*Triticum aestivum*)	- Increased ETH production	- Put decreased ETH production (through inhibition of ACC synthase)	- High level of ETH inhibited strong root elongation - Put restored root growth and mitigated stress	Yu et al. (2016)
Drought (*Hordeum vulgare*)	- Early ETH production - Triggered senescence	- Early Put production triggered the production of higher PAs (Spd and Spm)	- SAMDC induced tolerance by upregulating and promoting the creation of Spd and Spm and downregulating ETH synthesis	Montilla-Bascón et al. (2017)
Salt [*Capsicum annum* L. cv. Pairal, *Lactuca sativa* var. Longifolia Lam. cv. Inverna, *Spinacia oleracea* L. cv. Boeing, *Beta vulgaris* L. var. Crassa (Alef.) J. Helm. cv. Detroit]	- Sensitive plants: strong and prolonged ETH increase - Tolerant plants: moderate ETH increase	- Sensitive plants: early enhanced expression of PA enzymes - Tolerant plants: lower and delayed expression of PA enzymes	- Fresh weight, water content reduction was recorded the highest in sensitive species and the lowest in tolerant species	Zapata et al. (2017)
Drought (*Oryza sativa*)	- In moderate stress: ACS and ETH synthesis decreased - In severe stress: ACC and ETH stress increased	- In moderate stress: increased Spd and Spm - In severe stress: decreased Spd and Spm	- ETH and Spd/Spm interact antagonistically in response to stress. - In moderate stress: spikelet growth and grain output rose - In severe stress: sterility and degeneration increased.	Zhang et al. (2017)
Drought (*Oryza sativa*)	- In sensitive plants: ETH, ACC, and H_2_O_2_ decreased	- In sensitive plants: reduced PA synthesis	- In sensitive higher spikelet degradation	Chu et al. (2023)
Salt (*Zea mays*)	- Enhanced H_2_O_2_ production	- Increased *DAO* expression	In tolerant plants, salinity induced the expression and activity of *ACO*, leading to enhanced ETH biosynthesis, which induces H_2_O_2_ production due to enhanced *DAO* expression and activity. - H_2_O_2_ plays a dual role. It acts as a signalling molecule for PA synthesis during the first stage of stress - In the second stage of stress, H_2_O_2_ inhibits stress and ETH production and activates PA biosynthesis by upregulating *ADC* expression and activity	Freitas et al. (2018)
Ripening (*Fragaria ananassa*)	- Increased ETH production	- *FaSAMDC* overexpression increased Spm	- Put reduced fruit ripening - Spm enhanced ripening due to a synergistic combination of IAA, ABA, and ETH - *FaSAMDC* overexpression fruit ripening	Guo et al. (2018)
Urea application (*Oryza sativa*)	- Decreased ACC and ETH production	- Increased Spd and Spm	- Enzymes involved in amino acid production were linked to the PA and ACC/ETH ratios; as a result, the total amount of essential and non-essential amino acids rose.	Xu et al. (2022)
Salt (*Solanum lycopersicum*)	- Wild type: active ETH signalling and upregulated APX - ETH receptor mutant (*Nr*) mutants: impaired APX	- Wild type: PA synthesis increased - *Nr* mutant: PA synthesis and catabolism increased	- ETH and PAs acted synergistically	Takács et al. (2022)
Ripening (*Prunus persica*)	- ETH upregulated *PpeERF113* - *PpeERF113* activated *PpePAO1*	- Downregulated PA synthesis - Upregulated PA catabolism	- ETH-induced PA catabolism lowered PA levels, promoted ripening - PA buildup postponed ripening	Wang et al. (2022)

Fruit ripening is a genetically programmed and hormonally regulated phase of the plant. It is governed by several minerals and enzymes. ETH is a key enzyme in the ripening of climacteric fruits (Feder et al., 2020). It enhances processes such as increased respiration, cell wall disassembly, degradation of chlorophyll, biosynthesis of pigments, etc. and genes encoding ripening-related enzymes, including pectin methylesterase, expansins, polygalacturonase, and cellulose. Knockdown of the *ACS2* gene in tomatoes reduces flowering and fruit set and enlarges fruits (Sharma et al., 2021). Huang et al. (2025) reviewed ETH, and some TFs such as NAC, RIN, GH3, HD-ZIP, MADS-box, and helix-loop-helix are necessary for the initiation of ripening. ETH, together with PAs, regulates fruit ripening. It was found that Put works antagonistically with ETH, whilst higher PAs such as Spd and Spm work synergistically with ETH. Exogenous application of Spd on the young fruit of *Prunus persica* delayed ripening by impairing ETH signalling (Torrigiani et al., 2012; Gao et al., 2021). In addition, upregulation of the *FaSAMDC* gene speeds up fruit ripening and fruit anthocyanin, sugar, and hormone levels (ABA, ETH, and IAA), whilst Put and SAMDC inhibitors impede ripening (Guo et al., 2018). Chen et al. (2025) demonstrated that *JsSAMDCs* upregulate the genes of the biosynthesis of PAs, which regulate the flowering gene and promote female flower bud differentiation in *Juglans sigillata*. Wang et al. (2022) also studied the relationship of ETH–PA in fruit ripening. It was revealed that ETH directly upregulates PAO, namely, *PpeERF113* upregulates *PpePAO1*, which promoted tomato fruit ripening, just like overexpression of *PpePAO1* (**Figure 3**). Downregulation of *FaPAO5* is directly linked to the accumulation of Spd and Spm, which enhance ripening by regulating ripening-related events (Mo et al., 2020). Cao et al. (2022) also demonstrate that the copper amine oxidase (*CuAO*) gene (PA catabolism gene) induced under abiotic stress also promotes fruit ripening and reduces free PA content.

## 5 Conclusion and future perspectives

The complex interaction between ETH and PAs serves a vital regulatory role in plant growth, yield, stress response, and resilience. By understanding the interaction between these two, a revolutionary biochemical genetic network that controls plant growth, development, senescence, and adaptation to adverse environmental conditions can be revealed. Whether they interact antagonistically or synergistically, they influence plant growth and physiology by devising strategies to cope with environmental stresses. Hence, the dual function and feedback loop of these two allows researchers and agronomists to better use crop management and breeding techniques to maximise growth under unusual environmental circumstances.

Research should also focus on how SAM, a node between ETH and PA synthesis, influences synthesis and signalling under both normal and stressful environments. How the key players of the signalling pathway of ETH–PAs influence each other during abiotic stress will also be studied. As we aimed to highlight, new directions should be investigated in order to clarify the picture of these compounds. Furthermore, research will employ mutant and overexpression lines to provide deeper insight into the crosstalk of ETH–PAs, using several modern techniques such as genomics, transcriptomics, and metabolomics. ETH–PA interaction could be a promising option for advancing agriculture, including under stressful environment conditions. Their interaction provides a more resilient, sustainable, and predictive future; hence, crops not only endure but also flourish under stressful environments.
